# Relations of Diseases of the Eye to General Diseases

**Published:** 1895-09

**Authors:** 


RELATIONS OF DISEASES OF THE EYE TO GENERAL DISEASES. By MAX KNIES, M. D., Professor Extraordinary at the University of Freiburg. Forming a supplement volume to every manual and text-book of Practical Medicine and Ophthalmology. Edited by Henry D. Noyes, A. M., M. D., Professor of Ophthalmology and Otology in Bellevue Hospital Medical College; Executive Surgeon to the New York Eye and Ear Infirmary; recently President of the American Ophthalmological Society; recently Vice-President of the New York Academy of Medicine; Permanent Member of the New York State Medical Society: Member of the American Medical Association, etc., etc. Octavo, pp. x.—468. New York: William Wood & Co. 1895. Price, cloth, $4.25.

As a scholar and investigator, Prof. Knies has occupied high rank in ophthalmology for many years, and the publication of this masterpiece is hailed with gladness, both in its German and English dress. Very few works have issued from the medical press during the last decade that embody so much painstaking research as this. It covers a field not altogether new, but far from being fully explored. How well it has here been traversed can only be appreciated by a perusal of the volume itself.

The tendency of the profession today is to recognise more and more the intimate relation which exists between the eye and the system at large. That the ophthalmologist and the general practitioner and neurologist must join hands in diagnosis and therapeutics is more fully realised at the present time than ever before. The treatise before us is one of the most helpful contributions toward a proper understanding of ocular and systemic relations, both physiologically and pathologically, that has ever been made.

The book is divided into nine chapters, in which the author sets forth the relation of eye diseases to (1) diseases of the nervous system; (2) diseases of the skin; (3) diseases of the digestive organs; (4) diseases of the respiratory organs; (5) diseases of the circulatory organs; (6) diseases of urinary organs; (7) diseases of the sexual organs; (8) poisons and infectious diseases; (9) constitutional diseases. The first chapter deals with (*a*) the anatomical course of the nerves of the eye; (*b*) the disorders in the domain of the ocular nerves and their central origin; (*c*) the relations between the blood-vessels and the eye; (*d*) the relations between the eye and the lymphatics; and (*e*) individual diseases of the brain, cord and nerves. This chapter is, perhaps, the most interesting of all, and occupies 244 pages, or more than one-half of the book. In the second chapter the effects of erysipelas, eczema, herpes, lupus and other skin affections upon the eye are described. In chapter third, many eye affections are found to be due to diseases of the digestive apparatus, for example, of the teeth and intestines. Chapter fourth deals with diseases of the respiratory organs, including those of the nose and adjacent cavities, the ear and those of the respiratory tract proper. Diseases of the organs of circulation, treated in chapter fifth, furnish sufficient cause for retinal hemorrhages, inflammations, embolism and other fundus changes; while those of the urinary and sexual organs, dealt with in chapters sixth and seventh, reveal manifold sources of retinal disease and functional eye disturbances in various forms. The eighth and ninth chapters complete the work and occupy 130 pages. Here the general practitioner will find an immense fund of information regarding the effects of drugs and poisons on the eye, and the relation of infectious and constitutional diseases to diseases of the eye.

This most brief outline must suffice to indicate to the reader the extent of this work. It is a veritable storehouse of facts, citations and information, and embodies an invaluable wealth of material. Dr. Noyes has done the American profession an immense service in placing before it an English translation. No physician who desires to keep pace with progress of the age should fail to read it. A. A. H.

